# Asymptomatic stones in the lacrimal canaliculus

**DOI:** 10.1002/ccr3.8175

**Published:** 2023-11-22

**Authors:** Hongda Zhang, Maosheng Xu, Guanghao Qin, Jiayan Chen, Yifan Qi, Ling Xu, Wei He, Emmanuel Eric Pazo

**Affiliations:** ^1^ Department of Ophthalmology He Eye Specialist Hospital Shenyang China

**Keywords:** canaliculitis, dacryoliths, tear meniscus height

## Abstract

**Key Clinical Message:**

Asymptomatic lacrimal canaliculus stones causing many stones without symptoms are rare. The patient recovered well within a week after dacryolith removal. This diagnosis is prevalent in this age group. However, asymptomatic nasolacrimal obstruction should be considered.

**Abstract:**

Dacryoliths, also known as symptomatic stones, are frequently observed in the lacrimal drainage system. These stones manifest through symptoms such as conjunctivitis, discharge, and epiphora. Nevertheless, the occurrence of numerous stones in the lacrimal canaliculus, in the absence of apparent symptoms, is uncommon. In this study, we present a case with the presence of several stones within the inferior lacrimal canaliculus. A female patient, aged 74, appeared with bilateral senile cataracts and was scheduled for cataract surgery. During a standard ocular examination, it was observed that the tear meniscus height in the left eye had a greater magnitude compared with the right eye. Canaliculitis with dacryolith was verified using a series of diagnostic procedures, including physical inspection, fluorescent dye disappearance test, palpation, 50 Mhz ultrasound biomicroscope scan, and irrigation of the lacrimal canaliculi. Upon surgical investigation, the canaliculus obstruction was confirmed, characterized by the existence of many tiny dacryolith formations inside the inferior canalicular system. Following the surgical excision of the dacryoliths, the patient experienced a full remission within a week. While it is common for individuals in this age range to receive this diagnosis, it is important to consider silent nasolacrimal blockage as a potential alternative diagnosis. It is important to note that the presence of dacryoliths in the lacrimal drainage system might manifest independently of conjunctivitis. No discernible risk indicators were found in relation to the aforementioned patient.

## Graphical abstract

Unilateral lacrimal canalicular stones.

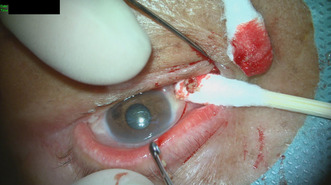

## 1 INTRODUCTION

Lacrimal canaliculitis can occur due to many causes, including epithelium or pericanalicular inflammation. Although certain causes, like herpetic canaliculitis, advance quickly, there are other causes that are more subtle and often remain unnoticed until the occurrence of epiphora.^1^, ^2^, ^3^, ^4^ Impaired function of both the active pumping mechanism and the static drainage of the lacrimal canaliculi can occur due to inflammation, scarring, and dacryoliths.^5^ The etiology of lacrimal sac dacryoliths remains uncertain; nevertheless, many writers have posited that the composition of dacryoliths may provide insights into their underlying cause.

**FIGURE 1:**
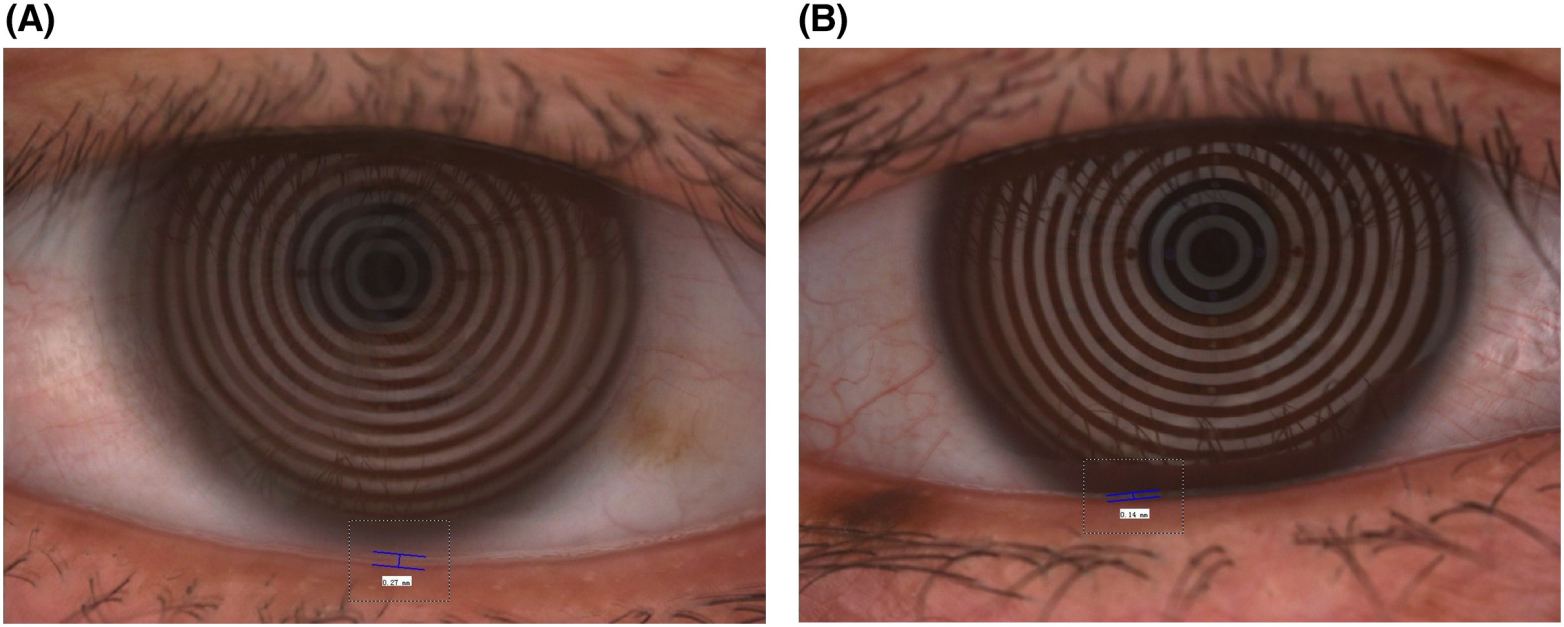
(A) OS: tear meniscus height measured by Keratograph 5M (Oculus). (B). OD: tear meniscus height measured by Keratograph 5M (Oculus).

**FIGURE 2:**
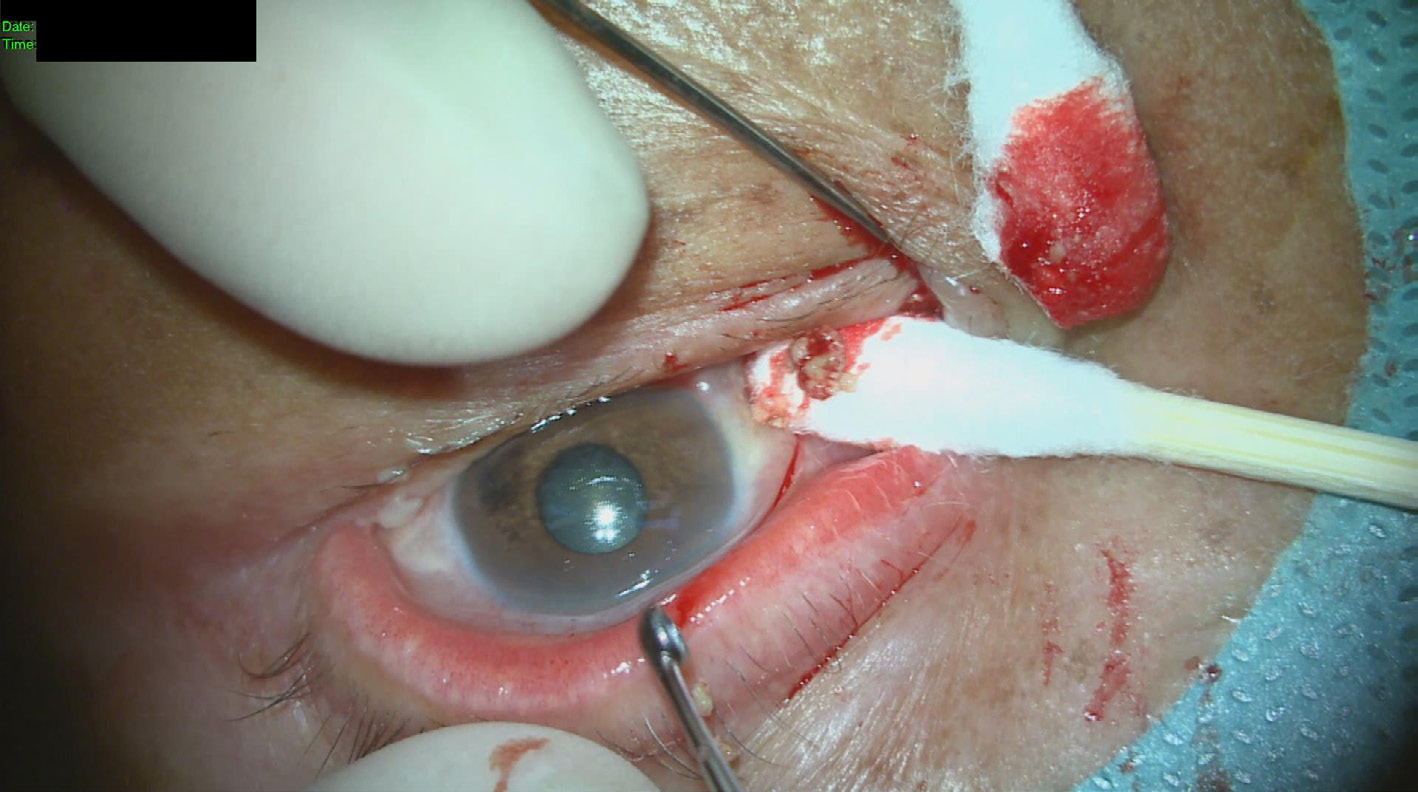
OS: Intraoperative presentation of the dacryolith in the inferior canalicular system.

A 74‐year‐old female patient appeared with bilateral senile cataracts and was scheduled for standard cataract surgery. The patient's medical background encompassed well‐managed diabetes and hypertension over a decade. The preoperative dry eye assessment conducted prior to cataract surgery involved the use of the Oculus Keratograph 5M device. The examination results indicated that the tear meniscus height (TMH) was higher in the left eye (27 mm) compared with the right eye (14 mm), as shown in Figure 1A,B. The observed difference in TMH between the two eyes led to a subsequent examination of the lacrimal canaliculus. It is worth mentioning that the tear break‐up time was seen to be within the range of normal values, specifically 8 s in the right eye and 10 s in the left eye. The results of the fluorescent dye disappearance test indicated the presence of a partial blockage in the left eye. There was an absence of conjunctivitis, epiphora, or purulent discharge. During the physical examination and palpation, a localized prominence was observed in the region of the left lower lacrimal canaliculus with no discomfort reported. The ultrasonic biomicroscope operating at a frequency of 50 MHz was able to identify an expanded lacrimal canalicular lumen and a tiny nonopaque structure in the region of the left lower lacrimal canaliculus.^6^ No presence of soft tissue mass or edema was seen. Following the acquisition of informed permission from the patient, a video recording of the surgical process was conducted intraoperatively, with the patient's signature confirming their agreement. The procedure involved the utilization of topical and local anesthesia administered to the left lower lacrimal canaliculi. A blunt needle was inserted, followed by routine procedures of probing, irrigation, and lacrimal system intubation. These interventions confirmed the presence of partial blockage. The lacrimal canaliculus was surgically opened, and a procedure known as lacrimal canalicular anastomosis was performed. Additionally, an artificial lacrimal duct was inserted following the removal of 52 little dacryoliths, each measuring approximately 0.06 × 0.06 × 0.07 cm in size (Figure 2).

## 2 PATHOLOGIC FINDINGS

A gross examination of the dacryolith revealed a single irregular piece of firm, yellowish‐brown material measuring approximately 0.06 × 0.06 × 0.07 cm (Figure 2) in dimension. Unfortunately, a microscopic evaluation of the stone was not performed due to the unavailability of the histology laboratory staff during this time.

## 3 DISCUSSION

Lacrimal canaliculitis is rare and varied; therefore, it typically goes misdiagnosed. Canaliculitis patients have epiphora, punctal discharge, and a pouting punctum, but they often misdiagnose it as chronic purulent conjunctivitis, hordeolum, chalazion, or dacryocystitis, resulting in unnecessary manipulations and delayed treatment.^7^ The average time between symptoms and diagnosis was 15 months (2 days–10 years) in Lin et al.'s study,^7^ compared with 6–36 months in other published findings,^7^ highlighting the challenge for doctors and socioeconomic burden.

Canaliculitis with or without dacryolith presents with erythema and swelling over the medial canthus, leading to epiphora and mucopurulent discharge.^8^ Actinomyces, a Gram‐positive rod, is the most frequent cause of canaliculitis. Dacryoliths usually lead to inflammation in the region and exhibit a female predominance.^9^ However, our patient had no inflammation or discharge. Probing and irrigation were performed since there were no apparent signs of lacrimal drain infections. In this case, we found partial obstruction. The patient had no history of punctal plug placement or use of systemic steroids and was not immunocompromised. Since 69% of canaliculitis and dacryocystitis are related to Propionibacterium infections, it may have been the causative agent in our patient.^10^

Alteration in tear fluid dynamics could have contributed to lacrimal canaliculi dacryolithogenesis. Tear stasis leads to an accumulation of debris and proteins, representing the stone's nidus. Therefore, particles of organic material such as hair or inorganic material may have led to tear stasis and, finally, stone formation.^2^

This case report is limited due to its follow‐up data and describes only a single case due to the rarity of this pathology and lack of radiologic imaging techniques.

Patients with lacrimal canaliculi dacryolith could be asymptomatic and might not present with typical signs of conjunctivitis, discharge, and epiphora. The diagnosis could be easily missed if TMH and fluorescent dye disappearance test is not performed. Physicians should consider lacrimal canaliculi dacryolith in the differential diagnosis of complete or partial obstruction of tear flow with increased TMH not presenting as epiphora. The following 2‐week post‐op revealed similar bilateral THM and no symptomatic complaints.

## AUTHOR CONTRIBUTIONS

**Hongda Zhang:** Conceptualization; investigation; methodology; validation; writing – original draft; writing – review and editing. **Maosheng Xu:** Project administration; writing – review and editing. **Guanghao Qin:** Methodology; project administration; writing – review and editing. **Jiayan Chen:** Software; visualization; writing – review and editing. **Yifan Qi:** Data curation; investigation; writing – review and editing. **Ling Xu:** Funding acquisition; project administration; resources; supervision. **Wei He:** Conceptualization; funding acquisition; project administration; resources; writing – review and editing. **Emmanuel Eric Pazo:** Conceptualization; formal analysis; methodology; supervision; validation; writing – review and editing.

## FUNDING INFORMATION

This study was entirely funded by He Eye Specialist Hospital, Shenyang, China. No support was received for the publication of this article.

## CONFLICT OF INTEREST STATEMENT

None.

## ETHICS STATEMENT

The study followed the Declaration of Helsinki and the He Eye Specialist Hospital, Shenyang, China, Institutional Review Board. Every research participant gave written informed permission. This study removes all personally identifiable data from the dataset, making it nonhuman subjects research.

## CONSENT

Written informed consent was obtained from the adult patient to publish this report in accordance with the journal's patient consent policy.

## Data Availability

Anonymized datasets generated and analyzed during the current study will be made available on reasonable request by the corresponding author (Emmanuel Eric Pazo, ericpazo@outlook.com).
